# Abstract of Proceedings of the Chicago Society of Physicians and Surgeons, for the Year 1873

**Published:** 1874-03

**Authors:** 


### ABSTRACT OF PROCEEDINGS OF THE CHICAGO SOCIETY OF PHYSICIANS AND SURGEONS, FOR THE YEAR 1873.

In connection with a recently published editorial on the subject of the duty of the medical profession to the medical societies of this city, we had intended to present a *resumé* of their transactions during the last year. In consequence of the loss of a portion of its record, we are unable to print an abstract of the proceedings of the Chicago Medical Society, in connection with that of the Chicago Society of Physicians and Surgeons, which is subjoined, though we have made an effort to do so, of whose result, had it been successful, we are assured.

It is not only gratifying to note an improvement in the character and value of these transactions, but they also present an appeal to the studious part of the profession, which cannot fail to meet with a hearty response.

I.—REPORTS.

1. Annual Report of Section on Practice of Medicine (one paper).

2. Annual Report of Section on Surgery (three papers: "A," * "Strictures of Urethra and Rectum;" "B," "Electro-Therapeutics of Surgery;" "C," * "Staphylorraphy and Uranoplasty"—with exhibition of gag and models).

3. Annual Report of Section on Pathology (two papers: "A," * "Pathology of Nervous System;" "B," "Pathology of Ovarian Tumors").

4. Annual Report of Section on Obstetrics and Diseases of Women and Children (two papers: "A," * "Diseases of Women;" "B," "Diseases of Children").

5. Annual Report of Officers of the Society.

6. * Report on the Literature of Cholera; with Abstracts from the Later Authorities.

7. * Report of Committee on the Prophylaxis, and Treatment of the Cholera Endemic, in 1873.

8. Report on the Therapeutic Value of Cosmoline.

II.—PAPERS.

1. On Centripetal and Centrifugal Neurosis.

2. * On Pathogenesis, as resulting from the use of Tobacco.

3. * On the Relations of the Integumentary and Generative Organs.

4. * On the use of Lime Vapor in Pseudo-Membranous Croup.

5. * On the Cervix Uteri, before, during, and after Labor (with drawings).

6. * On the Genetic Relations of the Marsh Fungi to Malarial Diseases (with exhibition of colored sketches, and microscopical preparations of *palmellæ*, sporules in human blood, saliva, etc).

7. * Review of Hall's Translation of Lanfranc's Surgery, published in London, in 1865.

8. * On the Physiological Relations of Alcohol.

9. On some Questions in Therapeutics.

10. * On the Waxy Kidney.

III.—REPORTS OF CASES.

1. Cases of Cholera, occurring near the Southern Limits of the City of Chicago, Endemic of 1873.

2. Additional Cases, in same general place and time (with exhibition of map of locality).

3. Additional Cases, in same locality, somewhat later.

4. Cases of Cholera in Louisville, in 1856.

5. Twelve Cases of Cholera in the City of Chicago, in 1873.

6. Case of Criminal Abortion, resulting in Death from Hæmorrhage.

7. Cases of Intra-laryngeal and Tracheal Medication.

8. * Case of Chronic Inflammation of Stomach, simulating Cancer (with Report of Necroscopy, and Microscopic Pathology, and exhibition of Stomach, in gross, with illuminated and magnified Sections).

9. Cases of Uterine Fibroids, relieved by the hypodermic injection of Ergotine.

10. Case of Foreign Body in the Urethra.

11. Case of Death from Gunshot Wound of the Cerebral Sinuses.

12. Case of intense Sciatic Pain from Suppressed Menstruation, relieved by the Thermo-Electric Bath.

13. Case of Destruction of the Epiglottis, from Syphilis, with retention of power of perfect Deglutition (with exhibition of patient).

IV.—SPECIMENS PRESENTED.

1. Semi-calcareous tumor, removed from the arm of pregnant female (with report of case).

2. Monster, with complete visceral ectrophy, four mammary glands, and no genito-urinary or intestinal apertures.

3. Heart, ruptured from muscular degeneration (with report of case).

4. Perforation of right auricle by pistol-ball (with report of case).

5. Ossified cardiac valves (with report of case).

6. Cancer of uterus (with report of case).

7. Cholera dejections (rice-water), mounted on microscopic slides, and illuminated (with report of case).

V.—EXHIBITIONS.

1. Anatomical models, purchased in Germany for the Chicago University.

2. Reproductions of photographs of cutaneous diseases, in colored crayon.

3. Models of invalid and operating chairs, close stools, etc.

4. Spectroscopic and other scientific apparatus.

Number of meetings during the year 1873, 22; total attendance at meetings, 436; average attendance at each meeting, 19.8.

* Papers thus marked, have appeared in the medical periodicals.

